# Pathogenicity of Novel H3 Avian Influenza Viruses in Chickens and Development of a Promising Vaccine

**DOI:** 10.3390/v17030288

**Published:** 2025-02-20

**Authors:** Shuning Zhou, Yaxin Zhang, Shuo Liu, Cheng Peng, Jiajing Shang, Jie Tian, Xiaoqi Li, Fuxiao Liu, Wenming Jiang, Hualei Liu

**Affiliations:** 1 College of Veterinary Medicine, Qingdao Agricultural University, Qingdao 266109, China; 2 China Animal Health and Epidemiology Center, Qingdao 266032, China; 3 College of Veterinary Medicine, Shanxi Agricultural University, Taiyuan 030801, China

**Keywords:** H3 avian influenza viruses, chicken, vaccine, protective effect

## Abstract

Since 2022, three cases of human infections of novel H3N8 avian influenza viruses (AIVs) have been confirmed in China. Given the potential for significant public health implications, the prompt detection and containment of the virus is particularly important. Comprehensive analyses were conducted of the complete viral gene sequences of five H3 subtype AIVs that were isolated from chickens, pigeons, and geese in live poultry markets in China in 2023. Four strains exhibited a high degree of homology with the H3N8 viruses responsible for human infections in 2022 and 2023. A subsequent study was conducted to investigate the pathogenicity differences among multiple subtypes of the H3 AIVs in chickens. The study revealed that all infected chickens exhibited clinical signs and viral shedding. Notably, two H3N8 viruses, which were highly homologous to human strains, demonstrated significant differences in adaptability to chickens. The goose-derived H3N5 strain displayed high adaptability to chickens and could replicate in multiple organs, with the highest titer in the cloaca. Additionally, a potential vaccine strain, designated CK/NT308/H3N3, was successfully developed that provided complete clinical protection and effectively prevented viral shedding against both H3N3 and H3N8 viruses. In conclusion, CK/NT308/H3N3 presents a promising vaccine candidate.

## 1. Introduction

Avian influenza viruses (AIVs) are RNA viruses that have undergone significant mutations and are classified within the genus *Alphainfluenzavirus* of the family *Orthomyxoviridae*. AIVs are classified based on the genetic diversity of the surface proteins hemagglutinin (HA) and neuraminidase (NA), which currently include 16 HA subtypes and nine NA subtypes [1]. Notably, the H5 and H7 subtypes, which can mutate from low to high pathogenicity, have caused severe outbreaks in poultry, resulting in the deaths of at least 422 million birds since 2005 [2]. The H9N2 and H3 AIV subtypes, which are low-pathogenicity strains, are often clinically asymptomatic or manifest as latent infections. Since 2010, the G57 H9N2 strain has become the most prevalent AIV strain in China. The AIV subtypes H5N6, H7N9, H10N3, and H10N8 can provide internal genes to recombinant strains and cause human infections [3]. The primary poultry hosts of H3 subtypes are waterfowl and are rarely isolated from chickens [4]. However, the recent identifications of novel recombinant H3N3 and H3N8 AIVs indicate adaptation and enhanced pathogenicity in chickens [5,6].

AIVs are predominantly found in wild birds and poultry [7]. However, there is an increasing prevalence of low-pathogenicity AIVs, including H3N2 and H9N2, in addition to the recently emerged H10N3 and H10N8 viruses, which have breached the interspecies barrier and caused human infections in China [8,9,10]. The H3 AIV subtypes have been isolated from a diverse range of mammals, including horses [11], dogs [12], pigs [13], and seals [14]. In April 2022, the first case of human infection of the H3N8 virus was documented in Henan Province, China [15]. Subsequently, another human infection case was reported in Hunan Province one month later in May 2022 [16]. Furthermore, the first human death attributed to the H3N8 virus occurred in 2023 [17], raising concerns about the potential for a pandemic. This suggests that H3 AIVs present a significant pandemic risk in human populations and that migratory birds play a crucial role in global dissemination. Consequently, the prevention and control of H3 AIVs have been prioritized as a critical global public health concern. Vaccination is the primary method for eradicating AIVs in poultry [18]. However, vaccination against the seasonal H3N2 virus does not produce cross-reactive antibodies that effectively target H3N8 viruses [19]. Therefore, the selection of vaccine strains with enhanced protective efficacy against H3 AIVs is particularly important to protect public health.

Here, five strains of H3 AIVs isolated from eastern China in 2023 were subjected to comprehensive analyses, including genomic characterization, infectivity testing in chickens, and vaccination-challenge experiments. The aim of this research was to enhance the understanding of the pathogenicity and vaccine efficacy of H3 viruses in chickens. The results of this investigation provide valuable insights to assess the threat posed by novel H3 AIV strains and to develop effective mitigation strategies.

## 2. Materials and Methods

### 2.1. Sample Collection and Description

Oropharyngeal and cloacal swabs, a total of 1224 in number, were collected from chickens, geese, and pigeons from live bird markets in three provinces of eastern China in 2023: Jiangsu, Jiangxi, and Guangdong. Among them, 954 swab samples were from chickens, 141 from geese, and 129 from pigeons. These swabs were suspended in 1 mL of sterile phosphate-buffered saline containing antibiotics (penicillin and streptomycin) and transported at 4 °C to China Animal Health and Epidemiology Center (Qingdao, China). Subsequently, the samples were inoculated into 10-day-old specific-pathogen-free (SPF) embryonated chicken eggs at a volume of 0.2 mL per embryo and incubated at 37 °C for 72 h. The virus strains identified as positive by hemagglutination assay (HA) and Sanger sequencing were purified through three rounds of the limiting dilution technique and stored at −80 °C. Notably, the isolates CK/P1027/H3N8, CK/G1106/H3N8, PG/J1247/H3N3, and GS/E1281/H3N5 had GISAID accession numbers 19689862–19689866, respectively, and were the focus of subsequent studies.

### 2.2. Genetic Sequence and Evolution

Phylogenetic analyses were performed to clarify the origins of these H3 subtype isolates. The Basic Local Alignment Search Tool (https://blast.ncbi.nlm.nih.gov/Blast.cgi, accessed on 9 October 2024) was used to align the complete viral genomes of the H3 isolates against sequences retrieved from the National Center for Biotechnology Information database (https://www.ncbi.nlm.nih.gov/, accessed on 9 October 2024) and the EpiFlu database of the Global Initiative on Sharing Avian Influenza Data (https://gisaid.org/, accessed on 9 October 2024). Sequences that matched the H3 isolates were subsequently downloaded. A phylogenetic tree of the eight gene segments was constructed utilizing Molecular Evolutionary Genetics Analysis software (version 11; https://www.megasoftware.net/, accessed on 9 October 2024) with the neighbor-joining method and bootstrap values based on 1000 repetitions. Additionally, complete sequences of the eight genes from the H3 isolates from human infections in China between 2022 and 2023 were incorporated into the phylogenetic tree to investigate the correlation between the isolates in this study and strains that originated from human cases.

### 2.3. Grouping of Animal Experiments

For this investigation, four-week-old SPF chickens were obtained from Beijing Boehringer Ingelheim Vital Biotechnology Co., Ltd. (Beijing, China), and randomly assigned to the control group, challenge group, or immune-challenge group. The control group consisted of 10 birds, while the immune-challenge and challenge groups were each subdivided into four subgroups of 10 birds, based on the strains being examined.

### 2.4. Chicken Infection Test

To investigate the reproductive capability and viral shedding potential of H3 viruses in chickens, five groups of four-week-old SPF chickens (*n* = 10/group) were established. One group served as the unchallenged control group, where chickens were kept without any virus inoculation. The remaining four groups were designated as challenge groups. Specifically, in each of these four challenge groups, chickens were intranasally inoculated with one of the four distinct isolates: CK/P1027/H3N8, CK/G1106/H3N8, PG/J1247/H3N3, or GS/E1281/H3N5. The inoculation was carried out using a 0.1 mL inoculum, and the virus dose within it was 10^6^ × the 50% embryo infectious dose (EID_50_). On days 3, 5, 7, 9, and 11 post-infection (dpi), oropharyngeal and cloacal swabs were collected from live chickens and inoculated into embryonated chicken eggs to calculate the 50% embryo infectious dose (EID_50_) for assessing virus shedding. On dpi 4, three chickens from each group were randomly euthanized and the trachea, lungs, Harderian glands, kidneys, spleen, cecum, pancreas, brain, thymus, heart, liver, and bursa of Fabricius were harvested for viral titration in eggs. The virus titer data in the bar chart were derived by calculating EID_50_ for each sample and averaging them for each challenge group. The tissues were stained with hematoxylin and eosin to identify lesions. After a 14-day observation period, the remaining chickens were euthanized, and blood samples were collected. The presence of specific antibodies in the serum was detected by the hemagglutination inhibition (HI) assay to assess seroconversion [20].

### 2.5. Vaccine Preparations

The strain A/chicken/China/NT308/2023(H3N3) (CK/NT308/H3N3) was selected to develop an effective vaccine strain against the H3 subtypes. As a promising vaccine candidate, this strain exhibited high homology to a human strain and produced a high titer in the hemagglutination test. This strain was propagated in the allantoic cavity of 10-day-old SPF chicken embryos at 37 °C for 72 h. The virus allantoic fluid (10^8^ EID_50_/0.1 mL) was then purified by centrifugation (20,000 rpm, 2 h, 4 °C) and inactivated with formaldehyde (*v*/*v* 0.2%, 2–3 days, 4 °C). Finally, the fully inactivated virus was mixed with a formulation of white oil No. 10 and Tween (23:40:1).

### 2.6. Vaccine Efficacy Trials

To evaluate the effectiveness of the vaccine, 40 four-week-old SPF chickens were subcutaneously immunized in the neck area with 10^8^ EID_50_ per 0.3 mL of the vaccine formulation, as the immune-challenge group. SPF chickens in the challenge group received an equivalent volume of phosphate-buffered saline. Three weeks post-vaccination, antibody titers were determined using the hemagglutination inhibition test with the vaccine strain (CK/NT308/H3N3) and three challenge virus strains (CK/P1027/H3N8, CK/G1106/H3N8, and PG/J1247/H3N3). Concurrently, each chicken (*n* = 10/group) was intranasally infected with CK/P1027/H3N8, CK/G1106/H3N8, PG/J1247/H3N3, or CK/NT308/H3N3 at a dose of 10^6^ EID_50_. Oropharyngeal and cloacal samples were collected on dpi 3, 5, 7, 9, and 11 to monitor virus shedding. Additionally, on dpi 4, organs from two randomly selected chickens were harvested, homogenized, and inoculated to titrate the virus and perform histopathological assessment.

## 3. Results

### 3.1. AIVs Isolation

The collected samples were inoculated into 10-day-old SPF embryos and five strains of AIVs were isolated. All eight gene fragments were sequenced with the Sanger sequencing method. In total, two H3N3 viruses, two H3N8 viruses, and one H3N5 virus were isolated, which were designated as A/chicken/China/P027/2023(H3N8)(CK/P1027/H3N8), A/chicken/China/G1106/2023(H3N8)(CK/G1106/H3N8), A/pigeon/China/J1247/2023(H3N3)(PG/J247/H3N3), A/goose/China/E1281/2023(H3N5)(GS/E1281/H3N5), and A/chicken/China/NT308/2023(H3N3)(CK/NT308/H3N3).

### 3.2. Phylogenetic and Genetic Analysis

**Figure 1:**
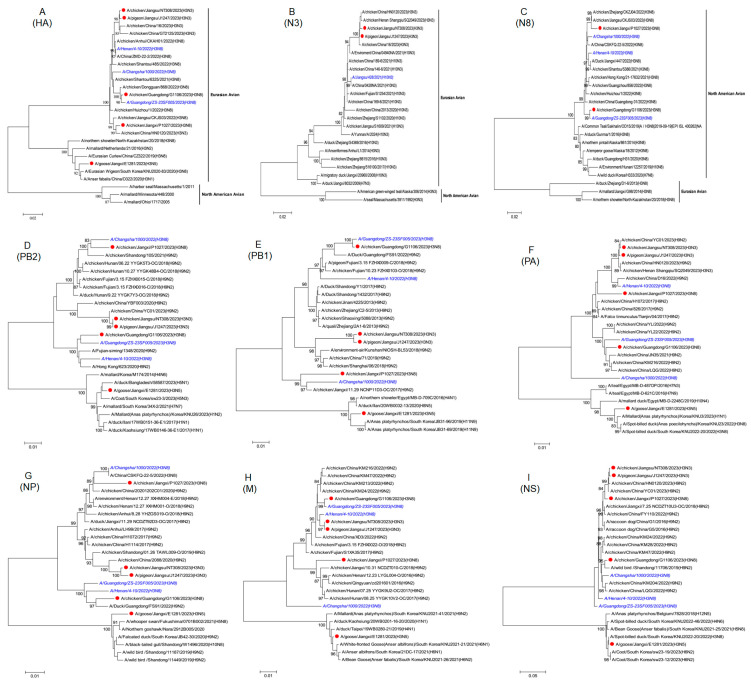
Phylogenetic trees illustrating the relationships among H3 AIV strains isolated in eastern China and the eight gene segments of human-derived strains. Phylogenetic trees of the H3, N3, and N8 genes are depicted in panels (**A**–**C**), respectively. The phylogenetic relationships among the PB2, PB1, and PA gene segments are illustrated in panels (**D**–**F**), respectively. Phylogenetic trees of the NP, M, and NS genes are provided in panels (**G**–**I**), respectively. The trees were constructed using the neighbor-joining method with MAGE 11 software with version 11.0.11. The strains isolated in this study are indicated by red solid circles, while AIVs identified as the cause of human infections in China are shown in blue italics. The strains marked in black were obtained from the Global Initiative on Sharing Avian Influenza Data and the Influenza Virus Resource of the National Center for Biotechnology Information. The scale represents the mean amino acid substitutions at each site.

The HA gene was determined to have originated in Eurasian avian species. These strains contained an amino acid motif (PEKQTR/GLF) or (PEKQTR/GIF) at the cleavage site between HA1 and HA2, indicating low-pathogenicity AIVs. All strains possessed 226Q and 228G residues (based on H3 numbering) at the receptor binding site, suggesting a preference for binding to SAα-2,3Gal avian-like receptors. The HA genes of CK/P1027/H3N8, CK/G1106/H3N8, PG/J1247/H3N3, and CK/NT308/H3N3 demonstrated high nucleotide homology with the H3N8 virus isolated from three infected humans in China, with homology levels ranging from 97.2% to 99.7% (Figure 1A). This finding suggests that the H3N8 virus responsible for human infections had originated in poultry. The phylogenetic tree indicates that the N8 genes of the two H3N8 strains examined were derived from North American avian species (Figure 1C). Furthermore, the N3 genes of the two N3N3 strains exhibited nucleotide homologies of up to 98.1% to 98.4% with the human isolate A/Jiangsu/428/2021 (H10N3) from China (Figure 1B). The internal genes of the five strains displayed considerable diversity, with the homology of the PB2, PB1, PA, NP, M, and NS genes ranging from 89.5% to 100% (Figure 1D), 87.9% to 99.6% (Figure 1E), 87.9% to 99.8% (Figure 1F), 89.4% to 99.5% (Figure 1G), 90.2% to 100% (Figure 1H), and 69.7% to 99.6% (Figure 1I), respectively. Among these strains, E1281/H3N5 formed a distinct branch, demonstrating a complex and diverse genetic origin. Notably, the internal genes of the remaining four strains were closely related to the G57 H9N2 virus and situated in the same clade as the isolates of infected humans.

### 3.3. Virulence and Replication of H3 Viruses in Chickens

**Figure 2:**
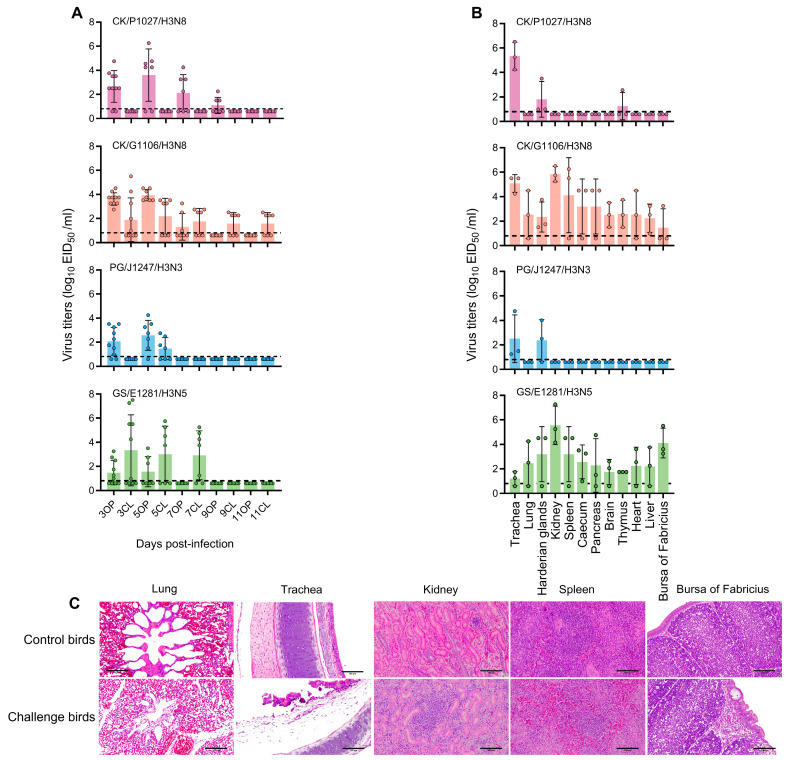
Pathogenicity of H3 subtype AIVs in chickens. (**A**,**B**) Shedding and replication of H3 virus in chickens. SPF chickens were inoculated with strains CK/P1027/H3N8, CK/G1106/H3N8, PG/J1247/H3N3, and GS/E1281/H3N5. Pharyngeal and cloacal swabs were collected at 2-day intervals from dpi 3 to 11, and the virus titer in eggs was determined (**A**). Concurrently, organ samples were collected on dpi 3, and the virus titer was determined in eggs (**B**). The columns represent the mean virus titer, while the error bars indicate the standard deviation. OP and CL refer to oropharyngeal and cloacal swabs, respectively. The dotted line on the *y*-axis indicates the lower limit of virus detection. Histological examinations of selected representative organs were conducted by the staining of microscopic sections with hematoxylin and eosin. From left to right, these included the lungs, trachea, and kidneys following infection with CK/G1106/H3N8, as well as the spleen and bursa of Fabricius following infection with GS/E1281/H3N5 (**C**).

During the 14-day observation period, all infected chickens exhibited a positive serum response with signs of depression and diarrhea, which persisted until dpi 7. Notably, there was a significant decrease (~30%) in food and water intake. All four strains were identified from the oropharyngeal swabs at dpi 3 and 5, with the highest titers observed at dpi 5 for CK/P1027/H3N8, CK/G1106/H3N8, and PG/J1247/H3N3, and at dpi 3 for GS/E1281/H3N5. The CK/G1106/H3N8 virus had the highest detection rate, while the SPF chickens demonstrated the longest detoxification period, lasting up to 11 days (Figure 2A). Interestingly, GS/E1281/H3N5 primarily underwent detoxification in the cloaca. Furthermore, CK/G1106/H3N8 and GS/E1281/H3N5 showed enhanced replication in chickens, with detection in multiple organs, including the trachea, lungs, Harderian glands, kidneys, spleen, cecum, pancreas, brain, thymus, heart, liver, and bursa of Fabricius on dpi 3. The trachea exhibited the highest titers of CK/P1027/H3N8, CK/G1106/H3N8, and PG/J1247/H3N3, while the highest viral titers of GS/E1281/H3N5 were detected in the kidneys, followed by the bursa of Fabricius (Figure 2B).

Histopathological examinations of the organs were conducted to gain insight into the histopathological alterations associated with H3 virus infection in chickens. The lungs, trachea, kidneys, spleen, and bursa of Fabricius from the four chickens exhibited a range of tissue damage, as illustrated in Figure 2C. The organs infected with CK/G1106/H3N8 displayed the most severe damage, including a reduction in alveolar space, an influx of red blood cells into the alveolar interstitium, the partial shedding of epithelial cells in the trachea, and significant infiltration of inflammatory cells into the white pulp of the spleen, accompanied by evidence of cell necrosis. Moreover, the kidneys of chickens infected with GS/E1281/H3N5 showed fibrosis and substantial inflammatory cell infiltration within the tubular interstitium. In comparison to the other three viruses, the bursa of Fabricius demonstrated structural incompleteness, with less distinct boundaries between the cortical and medullary regions.

### 3.4. Vaccine Protective Efficacy

**Figure 3:**
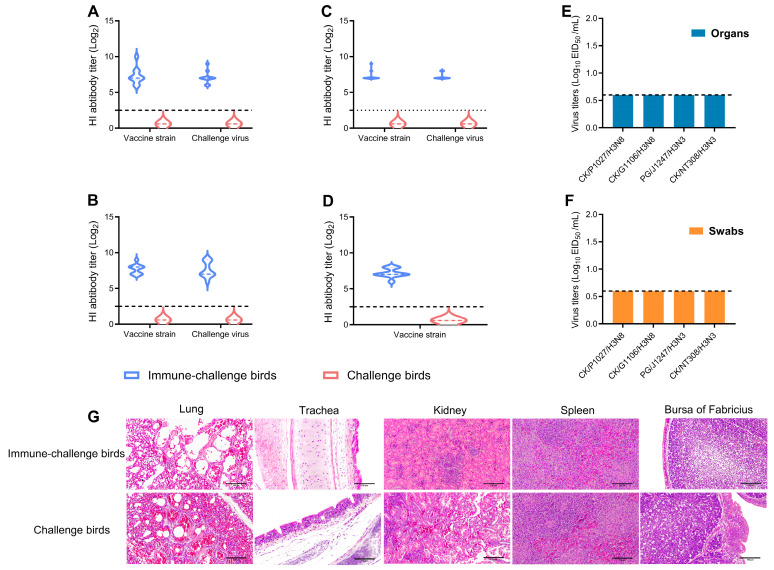
The level of immune protection provided by the vaccine. Antibody titers on dpi 21 with inactivated vaccines derived from the CK/NT308/H3N3 strain were assessed against the strains CK/P1027/H3N8 (**A**), CK/G1106/H3N8 (**B**), PG/J1247/H3N3 (**C**), and CK/NT308/H3N3 (**D**). On post-vaccination day 3, the viral titer (**E**) in eggs was quantified. Concurrently, oropharyngeal and cloacal swabs were obtained from the chickens on dpi 3, 5, 7, 9, and 11, and the viral titers (**F**) in eggs were determined. Microscopic sections of selected representative organs were stained with hematoxylin and eosin. From left to right, the examined organs included the lungs, trachea, kidneys, spleen, and bursa of Fabricius (**G**).

At 3 weeks post-vaccination, the antibody titer was quantified using the hemagglutination inhibition (HI) assay. The HI titers in chickens vaccinated with the inactivated vaccine derived from CK/NT308/H3N3 ranged from 7.20 ± 0.63 (log2) to 7.70 ± 0.67 (log2). In contrast, the antibody titer against the challenge virus demonstrated a decrease in only 0.10 log2, indicating that the vaccinated chickens were adequately protected. Conversely, the pre-challenge serum of the unvaccinated chickens showed no antibodies to the relevant strain (Figure 3A–D).

The absence of H3 virus detection (Figure 3E) suggests that the inactivated vaccine effectively inhibited the replication of H3 AIVs in all chicken organs. Furthermore, the lack of virus detection of the oropharyngeal and cloacal swabs (Figure 3F) indicates that the vaccine provided complete protection against virus shedding. The histopathological results revealed only minimal inflammation and cellular infiltration in the organs of the vaccinated chickens, demonstrating that the developed inactivated vaccine significantly attenuated pathological damage to the respiratory and digestive tracts.

## 4. Discussion

The recent emergence of the novel H3N8 virus has raised widespread public concern [21]. The two H3N8 viruses investigated in this study exhibited a high degree of similarity to the avian H3N8 viruses that have emerged over the past two years. The genome of this novel recombinant H3N8 virus comprises the H3 gene derived from Eurasian birds, the N8 gene derived from North American birds, and the internal genes of the G57 genotype H9N2 virus [22]. The novel H3N8 virus was initially detected in chicken flocks in July 2021 [23]. Prior to this study, the genetic origins of H3 AIVs have been reported in other countries. The M, NS, and PB2 gene fragments of the H3 strain isolated in South Korea are highly similar to previously isolated H9N2 viruses [24]. It is possible that H3 AIVs provided the internal genes for the highly pathogenic H5N8 virus [25]. A prior study demonstrated that the N6 and M genes of the distinct H3 strains exhibited a close evolutionary relationship with highly pathogenic H5 strains [26]. The novel H3N8 virus may have originated from either the H5N8 or H10N8 strains [27].

The PG/J1247/H3N3 virus isolated from pigeons showed poor adaptation in chickens. In contrast, a strain of the same subtype previously reported by our team demonstrated effective replication in chickens [5]. Experimental studies indicated that the H3 virus isolated from ducks predominantly replicated in the upper respiratory tract of chickens [28], thereby confirming the influence of the host on the pathogenicity of the virus. Notably, two H3N8 viruses carrying the H9N2 internal gene exhibited disparate infectivity in chickens. The CK/G1106/H3N8 strain exhibited increased pathogenicity and the ability to replicate in multiple organs of chickens, in agreement with the results of a previous study [6]. Conversely, CK/P1027/H3N8 primarily replicates in the upper respiratory tract of chickens. The reason for the discrepancy in replication kinetics between the genetically similar isolates CK/P1027/H3N8 and CK/G1106/H3N8 remains unclear, thus warranting further investigations. The H3N5 strain under investigation was predominantly excreted through the cloaca, consistent with the findings of a previous report [29]. Wild birds serve as natural hosts of AIVs [30]. The H3N8 virus is not readily transmissible from wild birds to commercial chickens [31] but can effectively replicate in SPF chickens [28]. The continued transmission and evolution of AIVs in chickens may increase the risk for human infections.

The emergence of the novel H3N8 virus in humans has been reported in China, raising concerns about the potential for a pandemic. H3N8 isolates from seals have demonstrated efficient replication in human lung cells [14]. Furthermore, human H3N8 isolates have been shown to effectively replicate in human respiratory epithelial cells [17] and are transmitted between ferrets via airborne routes [23]. The continued spread of the H3N8 virus may pose a threat to both poultry farms and humans. Hence, the implementation of a vaccination program is a potential solution. Since December 1994, the government of Mexico has deployed 8.47 billion doses of inactivated vaccines to control outbreaks of the H5N2 virus [32]. Inoculation with the H5/H7 bivalent inactivated vaccine reduced the detection rate of the H7N9 virus in poultry by 93.3% [33]. The H9N2 subtype is the most prevalent AIV worldwide [34]. Vaccination is a common method to control the spread of the H9 subtype [35]. Since 1998, approximately 500 million doses of the inactivated H9N2 vaccine have been administered annually to poultry in China [36]. An inactivated H9N2 whole-virus vaccine can effectively mitigate the clinical manifestations observed in immunized chickens [37]. While other forms of AIV vaccines, including DNA vaccines [38] and a novel live vaccine vector against the Newcastle disease virus [39], have been developed, inactivated whole-virus vaccines remain the predominant method of vaccination [40]. Vaccination may also serve as an effective strategy to prevent and control the spread of H3 viruses. In the present study, the antibody titer of chickens immunized with the inactivated vaccine produced from the CK/NT308/H3N3 isolate exceeded 7 log_2_ and provided 100% protection against shedding, suggesting that the vaccine is a promising candidate for further development.

Currently, despite the sporadic nature of human infections, it is essential to maintain vigilance against potential, as H3 viruses continue to evolve and may recombine with the seasonal H3N2 subtype. Therefore, the active surveillance of H3 viruses and the selection of appropriate vaccine strains are critical for pandemic preparedness.

## Data Availability

The data supporting this study’s findings are available from the corresponding author upon rea-sonable request.
